# Author Correction: Tracing immune cells around biomaterials with spatial anchors during large-scale wound regeneration

**DOI:** 10.1038/s41467-023-42118-4

**Published:** 2023-10-06

**Authors:** Yang Yang, Chenyu Chu, Li Liu, Chenbing Wang, Chen Hu, Shengan Rung, Yi Man, Yili Qu

**Affiliations:** 1 Department of Oral Implantology & State Key Laboratory of Oral Diseases and National Clinical Research Center for Oral Diseases, West China Hospital of Stomatology, Sichuan University, Chengdu, 610041 China; 2 Department of Prosthodontics & State Key Laboratory of Oral Diseases and National Clinical Research Center for Oral Diseases, West China Hospital of Stomatology, Sichuan University, Chengdu, 610041 China

**Keywords:** Gene expression analysis, Lymphocyte activation, Biomedical materials, Biomedical materials

Correction to: *Nature Communications* 10.1038/s41467-023-41608-9, published online 26 September 2023

The original version of this Article contained an error in Figure 1j, in which the left bar graph was mistakenly duplicated with the right bar graph. This has been corrected in both the PDF and HTML versions of the Article.

