# Supplementary material for: Tracing immune cells around biomaterials with spatial anchors during large-scale wound regeneration
Source: Nat Commun. 2023 Sep 26;14:5995. doi: 10.1038/s41467-023-41608-9 (PMC10522601; doi:10.1038/s41467-023-41608-9)
Supplement: Supplementary file 1 — Supplementary Information [file 41467_2023_41608_MOESM1_ESM.pdf]

Supplementary information:

Tracing immune cells around biomaterials with spatial anchors during large-scale wound regeneration

Yang Yang[12]†, Chenyu Chu[1]†, Li Liu[1]†, Chenbing Wang[1], Chen Hu[1], Shengan Rung[12], Yi Man[1]*, Yili Qu[2]*

[1] Department of Oral Implantology & State Key Laboratory of Oral Diseases and National Clinical Research Center for Oral Diseases, West China Hospital of Stomatology, Sichuan University; Chengdu 610041, China.

[2] Department of Prosthodontics & State Key Laboratory of Oral Diseases and National Clinical Research Center for Oral Diseases, West China Hospital of Stomatology, Sichuan University; Chengdu 610041, China.

* Corresponding author. Email: manyi780203@126.com (Y.M.) & qqyili@126.com (Y.Q.)

† These authors contributed equally to this work

* These authors jointly supervised this work

This PDF file includes:

Supplementary Fig. 1 Evaluation of the healing process of splinted and unsplinted wounds.

Supplementary Fig. 2 Analysis of Ctrl_LW and ECM_LW samples.

Supplementary Fig. 3 Gating scheme for T cells and macrophages.

Supplementary Fig. 4 Overview of the single-cell transcriptome analysis between ECM_LW and Ctrl_LW.

Supplementary Fig. 5 Further analysis of fibroblasts and T cells between ECM_LW and Ctrl_LW.

Supplementary Fig. 6 Subclustering analysis of neutrophils and dendritic cells between ECM_LW and Ctrl_LW

Supplementary Fig. 7 Histological analysis of WT and Rag2$^{-/-}$ mice treated with biomaterials.

Supplementary Fig. 8 Overview of the single-cell transcriptome analysis between WT and Rag2$^{-/-}$ mice treated with biomaterials.

Supplementary Fig. 9 Subclustering analysis of neutrophils and dendritic cells between WT and Rag2$^{-/-}$ mice.

Supplementary Figures

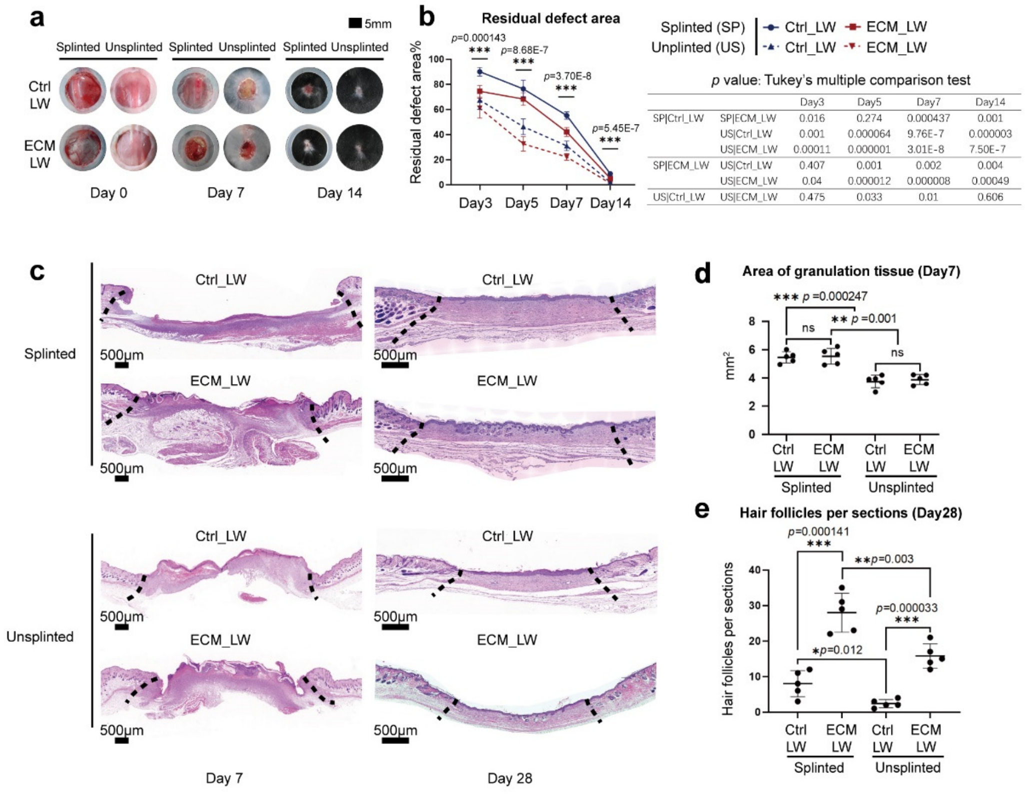

Supplementary Fig. 1 Evaluation of the healing process of splinted and unsplinted wounds. (a) Residual wound area of splinted and unsplinted wounds treated with saline or ECM scaffolds, and (b) corresponding analysis (Data are presented as mean ± SD, n=4 biologically independent samples, one-way ANOVA with Tukey's multiple comparison test, *p* values shown in the figure). (c) Representative H&E images of each group on POD 7 and POD28. (d) Quantitative evaluation of the area of granulation tissue (Data are presented as mean ± SD, n=5 biologically independent samples, two-tailed t-test, *p* values shown in the figure). (e) Histologic quantification of de novo HFs on POD28 (Data are presented as mean ± SD, n=5 biologically independent samples, two-tailed t-test, *p* values shown in the figure). *p* value: $*p < 0.05$, $**p < 0.01$, $***p < 0.001$, and $****p < 0.0001$.

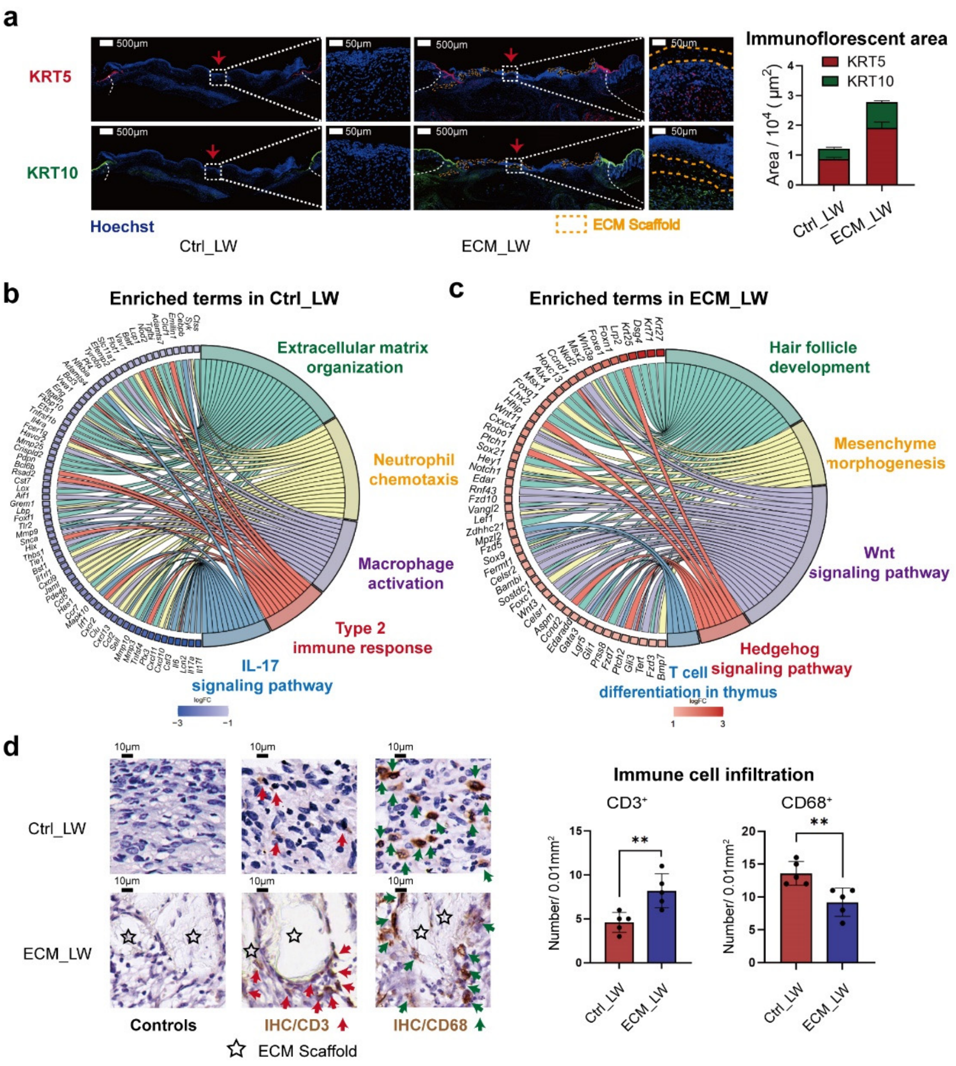

Supplementary Fig. 2 Analysis of Ctrl_LW and ECM_LW samples. (a) Representative IF images of IFEB[1] using KRT5 (red) and IFED[1] using KRT10 (green) on POD7 and semiquantitative evaluation of the fluorescent area (Data are presented as mean ± SD, n=4 biologically independent samples, KRT5: two-tailed t'-test, $^{**}p = 0.001$; KRT10: two-tailed t-test $^{****}p = 0.000008$). (b-c) Chord plots of enriched terms in Ctrl_LW group (b) and ECM_LW group (c). (d) Representative IHC images of stained T cells ($CD3^{+}$) and monocyte-macrophages ($CD68^{+}$) on POD7 and corresponding quantitative analysis (Data are presented as mean ± SD, n=5 biologically independent samples, two-tailed t-test, CD3 $^{**}p = 0.007$; CD68 $^{**}p = 0.008$). p value: $^{*}p < 0.05$, $^{**}p < 0.01$, $^{***}p < 0.001$, and $^{****}p < 0.0001$.

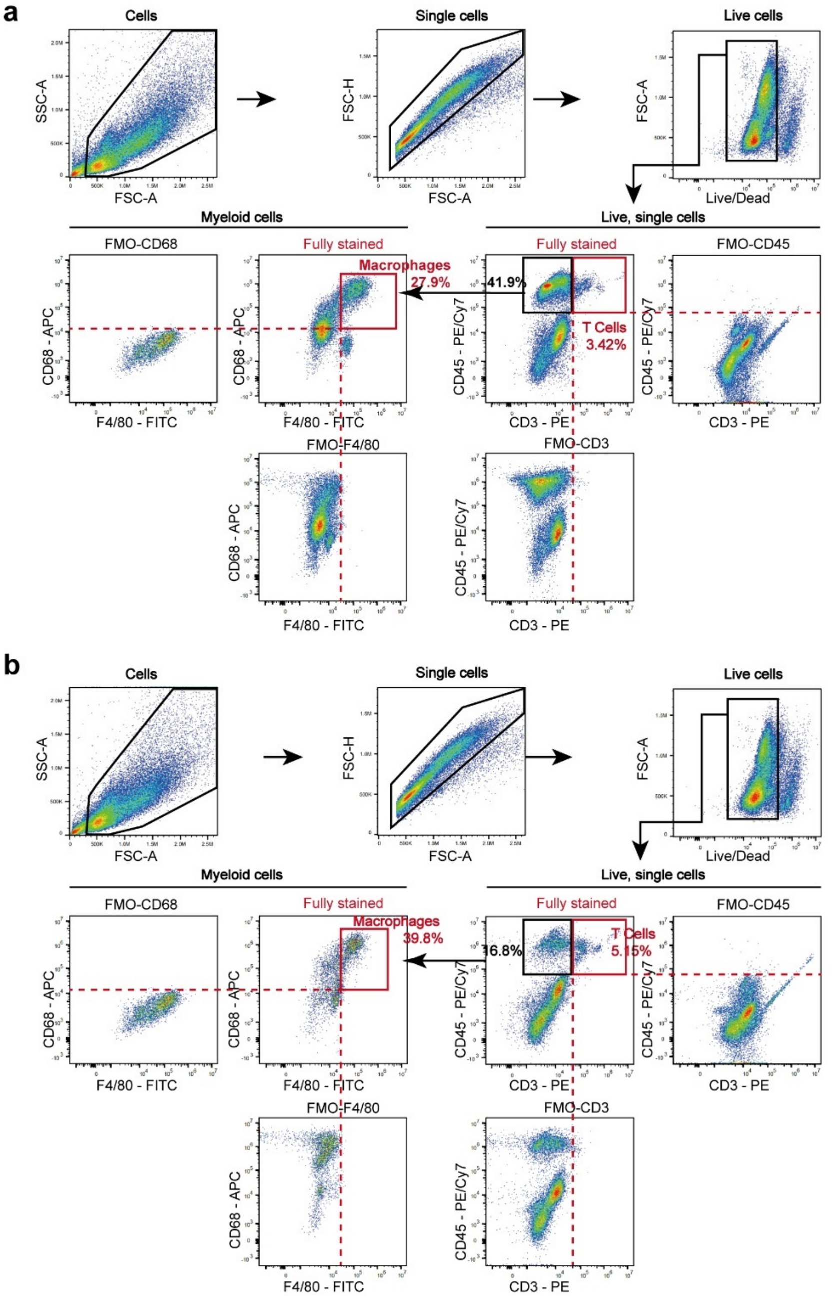

Supplementary Fig. 3 Gating scheme for T cells and macrophages. Multicolor flow cytometry gating strategy to isolate T cells ($CD45^+CD3^+$) and macrophages ($CD45^+CD3^-$ $CD68^+F4/80^+$) from single cell suspension. Representative flow cytometry plots of Ctrl_LW (a) and ECM_LW (b) samples on POD7. Abbreviation: FMO, Fluorescence Minus One.

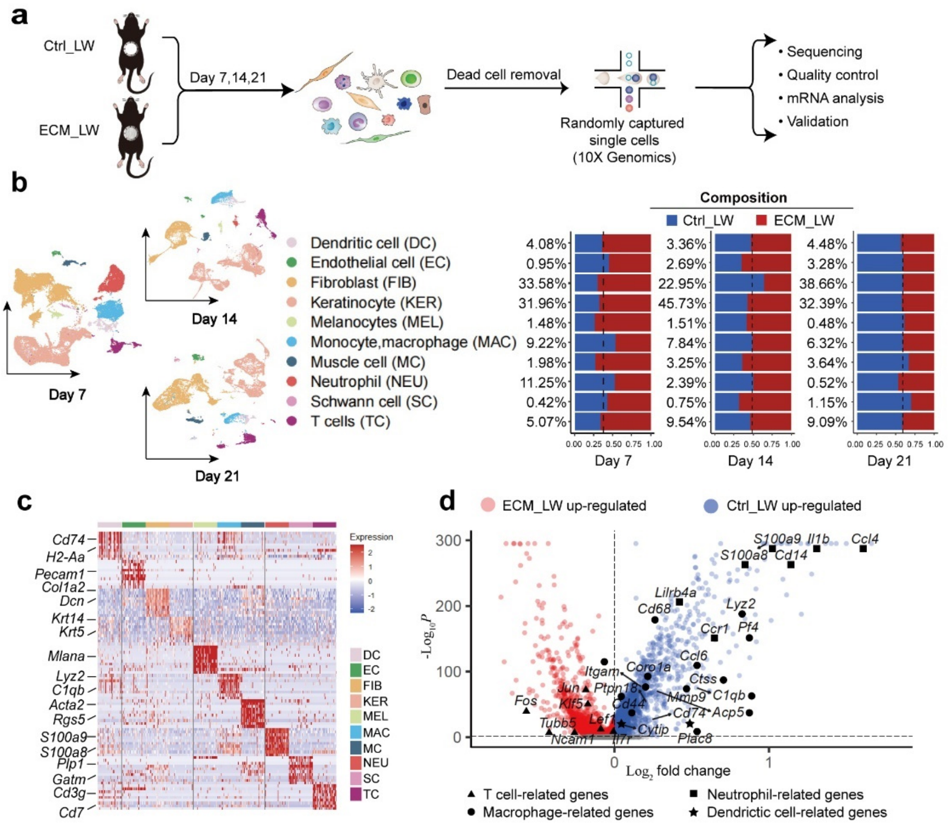

Supplementary Fig. 4 Overview of the single-cell transcriptome analysis between ECM_LW and Ctrl_LW. (a) Single-cell experiment workflow. (b) Cells are categorized into 10 main clusters. The number of cell populations in each cluster, number of cells (%), and composition of ECM_LW and Ctrl_LW groups are listed. (c) Heatmap showing top 10 marker genes for each cluster. (d) Volcano plot showing interested differentially expressed genes in ECM_LW and Ctrl_LW group (day 7).

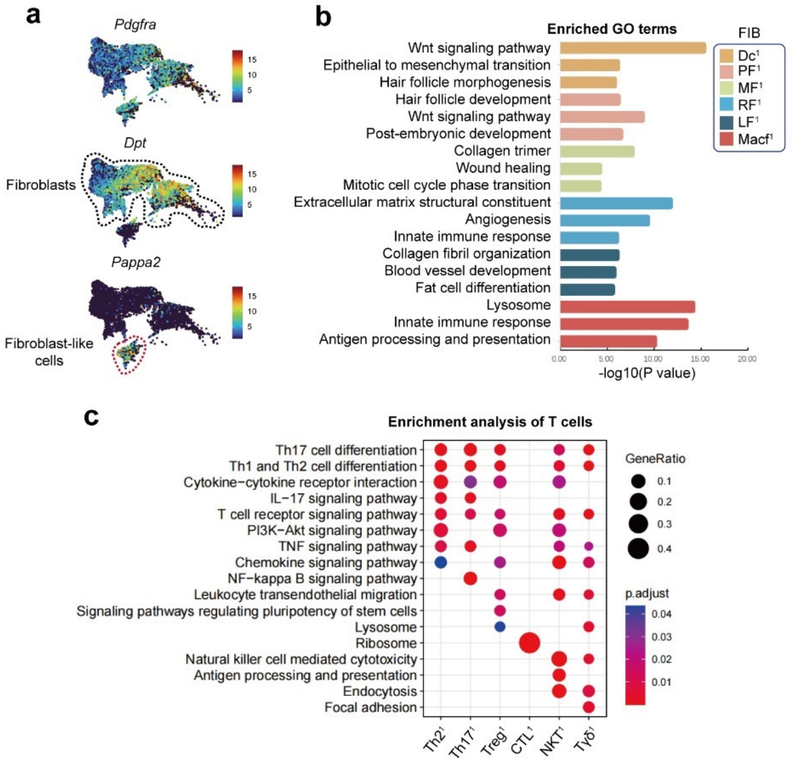

Supplementary Fig. 5 Further analysis of fibroblasts and T cells between ECM_LW and Ctrl_LW. (a) Feature plot of the marker genes of pan-fibroblasts (*Pdgfra),* fibroblasts (*Dpt*), and fibroblast-like cells (*Pappa2)*. (b) GO enrichment analysis of fibroblast subtypes. (c) Enrichment analysis of T cell subtypes.

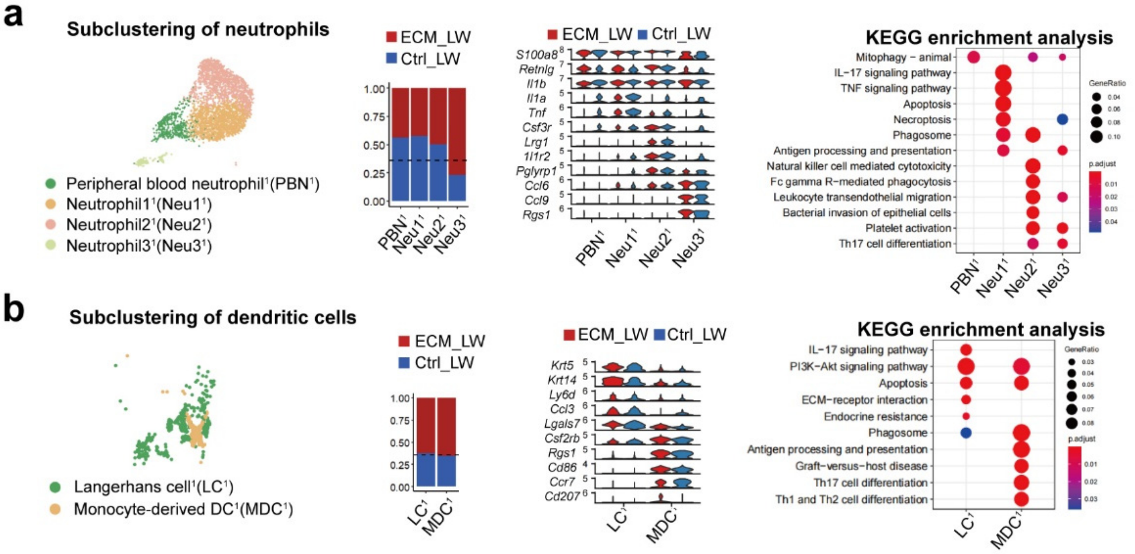

Supplementary Fig. 6 Subclustering analysis of neutrophils and dendritic cells between ECM_LW and Ctrl_LW. (a) Subclustering of neutrophils showing four subsets. The marker genes, composition, and KEGG enrichment analysis for each subset are listed. (b) Subclustering of dendritic cells showing two subsets, marker genes, composition, and KEGG enrichment analysis are listed.

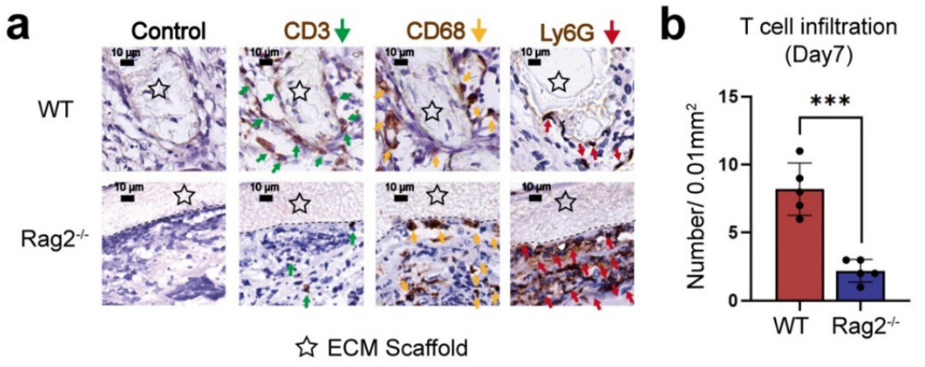

Supplementary Fig. 7 Histological analysis of WT and Rag2$^{-/-}$ mice treated with biomaterials. (a) IHC staining of CD3, CD68, and Ly6G. (b) semi-quantification of CD3^{+} T cell infiltration (Data are presented as mean ± SD , n=5 biologically independent samples, two-tailed t-test, ***p = 0.000210). p value: *$p < 0.05$, **$p < 0.01$, ***$p < 0.001$, and ****$p < 0.0001$.

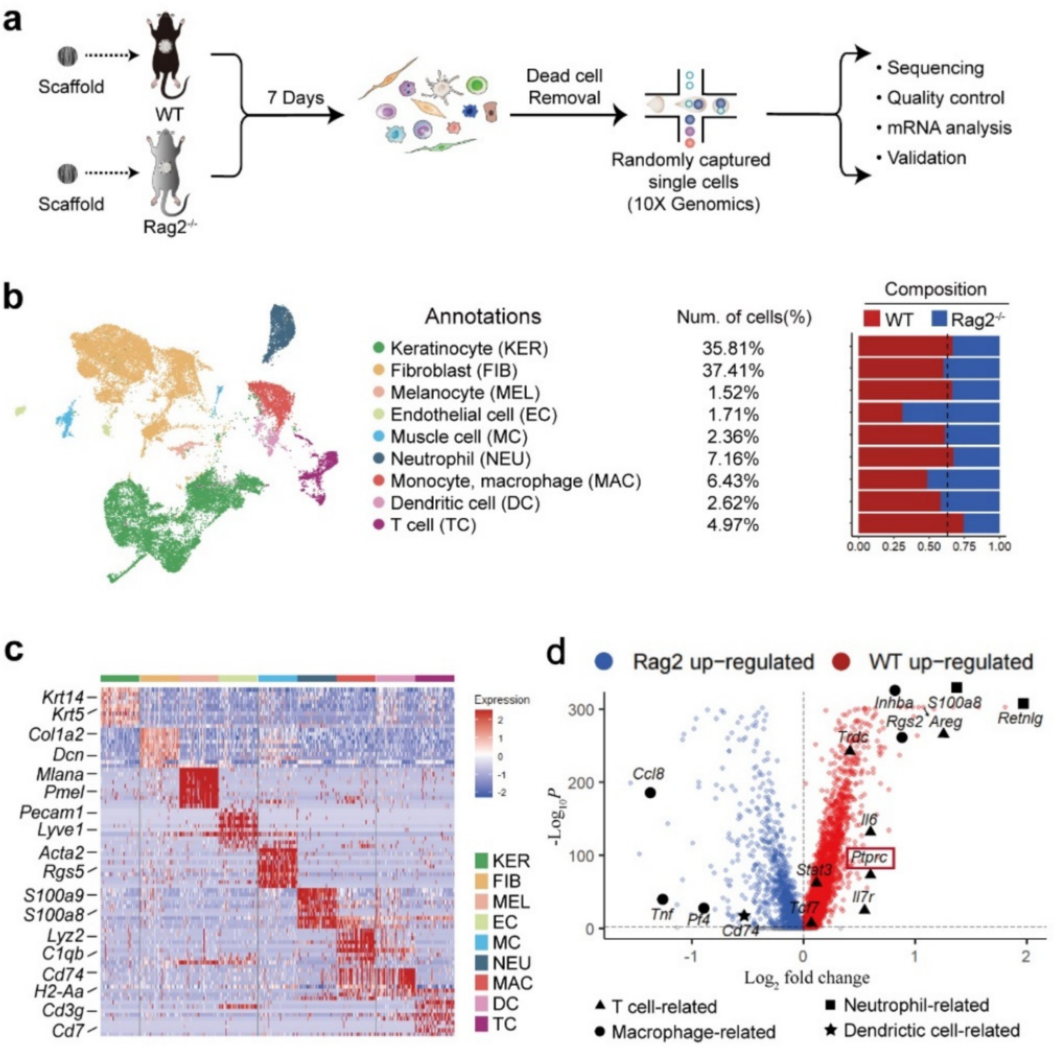

Supplementary Fig. 8 Overview of the single-cell transcriptome analysis between WT and Rag2$^{-/-}$ mice treated with biomaterials. (a) Single-cell experiment workflow. (b) Cells are categorized into nine main clusters. The number of cell populations in each cluster, number of cells (%), and composition of ECM_LW and Ctrl_LW groups are listed. (c) Heatmap showing the top 10 marker genes for each cluster. (d) Volcano plot showing interested differentially expressed genes in WT and Rag2$^{-/-}$ group (scRNA-seq).

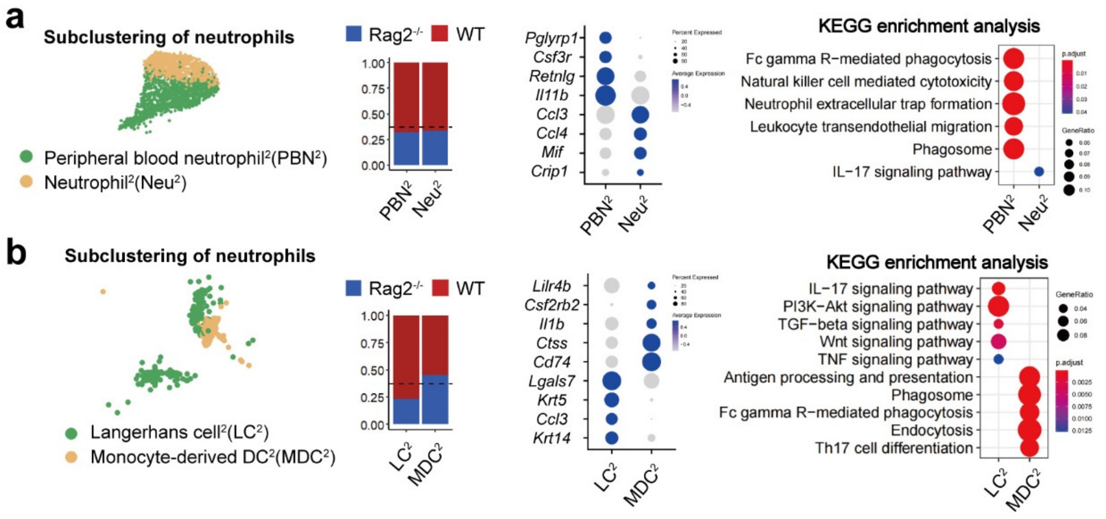

Supplementary Fig. 9 Subclustering analysis of neutrophils and dendritic cells between WT and Rag2$^{-/-}$ mice. (a) Subclustering of neutrophils showing two subsets. The marker genes, composition, and KEGG enrichment analysis for each subset are listed. (b) Subclustering of dendritic cells showing two subsets, marker genes, composition, and KEGG enrichment analysis are listed.
