# Supplementary material for: Tracing immune cells around biomaterials with spatial anchors during large-scale wound regeneration
Source: Nat Commun. 2023 Sep 26;14:5995. doi: 10.1038/s41467-023-41608-9 (PMC10522601; doi:10.1038/s41467-023-41608-9)
Supplement: Supplementary file 3 — Reporting Summary [file 41467_2023_41608_MOESM3_ESM.pdf]

Corresponding author(s): Yili Qu

Last updated by author(s): Aug 14, 2023

Reporting Summary

Nature Portfolio wishes to improve the reproducibility of the work that we publish. This form provides structure for consistency and transparency in reporting. For further information on Nature Portfolio policies, see our Editorial Policies and the Editorial Policy Checklist.

Statistics

For all statistical analyses, confirm that the following items are present in the figure legend, table legend, main text, or Methods section.

n/a	Confirmed	
☐	☒	The exact sample size (n) for each experimental group/condition, given as a discrete number and unit of measurement
☐	☒	A statement on whether measurements were taken from distinct samples or whether the same sample was measured repeatedly
☐	☒	The statistical test(s) used AND whether they are one- or two-sided<br>*Only common tests should be described solely by name; describe more complex techniques in the Methods section.*
☒	☐	A description of all covariates tested
☐	☒	A description of any assumptions or corrections, such as tests of normality and adjustment for multiple comparisons
☐	☒	A full description of the statistical parameters including central tendency (e.g. means) or other basic estimates (e.g. regression coefficient) AND variation (e.g. standard deviation) or associated estimates of uncertainty (e.g. confidence intervals)
☐	☒	For null hypothesis testing, the test statistic (e.g. F, t, r) with confidence intervals, effect sizes, degrees of freedom and P value noted<br>*Give P values as exact values whenever suitable.*
☒	☐	For Bayesian analysis, information on the choice of priors and Markov chain Monte Carlo settings
☒	☐	For hierarchical and complex designs, identification of the appropriate level for tests and full reporting of outcomes
☐	☒	Estimates of effect sizes (e.g. Cohen's d, Pearson's r), indicating how they were calculated

Our web collection on statistics for biologists contains articles on many of the points above.

Software and code

Policy information about availability of computer code

Data collection	Cell Ranger (v3.0.0) and Space Ranger (v2.0.0) were used to generate output of single cell RNA sequencing data and spatial transcriptomics data.
Data analysis	All R packages were used with R v4.1.0 with the most up-to-date packages available. R packages used : Seurat(v4.1.0), harmony (v0.1.1), clustree (v0.5.0), tidyverse (v2.0.0), Matrix (v2.0.0), ggplot2 (v3.4.2), DEseq2 (v1.32.0), GOplot (v1.0.2), reshape2 (v1.4.4), stringr (v1.5.0), EnhancedVolcano (v1.10.0), ClusterProfiler (v4.6.2), org.Mm.eg.db (v3.13.0), Cellchat (v1.5.0), patchwork (v1.1.2), data.table (v1.14.8), hdf5r (v1.3.8), pracma (v2.4.2). The Seurat software is available at https://satijalab.org/seurat/index.html. The ClusterProfiler software is available at https://github.com/YuLab-SMU/clusterProfiler. The GOplot software is available at https://github.com/cran/GOplot. The EnhancedVolcano software is https://github.com/kevinblighe/EnhancedVolcano. The Cellchat software is available at https://github.com/sqjin/CellChat.

For manuscripts utilizing custom algorithms or software that are central to the research but not yet described in published literature, software must be made available to editors and reviewers. We strongly encourage code deposition in a community repository (e.g. GitHub). See the Nature Portfolio guidelines for submitting code & software for further information.

Data

Policy information about availability of data

All manuscripts must include a data availability statement. This statement should provide the following information, where applicable:

- Accession codes, unique identifiers, or web links for publicly available datasets
- A description of any restrictions on data availability
- For clinical datasets or third party data, please ensure that the statement adheres to our policy

All data supporting the findings from this study are available within the article and supplementary files and from the corresponding author upon reasonable request. The RNA sequencing data used in this study are available in the NCBI Sequence Read Archive (SRA) database under accession code: PRJNA1004430. Source data are provided in this paper.

Human research participants

Policy information about studies involving human research participants and Sex and Gender in Research.

Reporting on sex and gender	NA
Population characteristics	NA
Recruitment	NA
Ethics oversight	NA

Note that full information on the approval of the study protocol must also be provided in the manuscript.

Field-specific reporting

Please select the one below that is the best fit for your research. If you are not sure, read the appropriate sections before making your selection.

☒ Life sciences ☐ Behavioural & social sciences ☐ Ecological, evolutionary & environmental sciences

For a reference copy of the document with all sections, see nature.com/documents/nr-reporting-summary-flat.pdf

Life sciences study design

All studies must disclose on these points even when the disclosure is negative.

Sample size	Sample sizes were determined on the basis of 10X Genomics protocols and previous uses of the model (Hu et al., 2021, Science Advances). For in-vivo experiments, n defines the number of animal used per condition. We have mentioned the exact value of n in the respective figure legends.
Data exclusions	Cell and gene filtering based on expression and accessibility values were performed.
Replication	Attempts at data replication were successful. Most of our experiments are replicated at least three times. The exact number of technical and/or biological replicates are reflected in the figure legends and the 'Statistics and reproducibility' section .
Randomization	The animals were randomly allocated to experimental groups.
Blinding	The experiments were performed taking into consideration an ethical and reductionist approach of animal usage. Animal studies were not performed in a blinded fashion. Since single cells were analyzed in an unbiased way and the conclusions were based on quantitative, objective gene expression data.

Reporting for specific materials, systems and methods

We require information from authors about some types of materials, experimental systems and methods used in many studies. Here, indicate whether each material, system or method listed is relevant to your study. If you are not sure if a list item applies to your research, read the appropriate section before selecting a response.

Materials & experimental systems

n/a	Involved in the study	
☐	☒	Antibodies
☒	☐	Eukaryotic cell lines
☒	☐	Palaeontology and archaeology
☐	☒	Animals and other organisms
☒	☐	Clinical data
☒	☐	Dual use research of concern

Methods

n/a	Involved in the study	
☒	☐	ChIP-seq
☐	☒	Flow cytometry
☒	☐	MRI-based neuroimaging

Antibodies

Antibodies used

Immunofluorescence staining:
cytokeratin 5 , ab52635, Abcam ,
cytokeratin 10 , ab76318, Abcam,
cytokeratin 17 , 17516-1-AP, Proteintech,
TWIST2, 66544-1-Ig, Proteintech ,
Ki67, Servicebio, GB121141,
SCD1, 28678-1-AP, Proteintech,
CRABP1, 13163S, Cell Signaling,
MEST, 11118-1-AP, Proteintech,
F4/80, 29414-1-AP, Proteintech,
CD206, 360017, Zenbio,
FOXP3 sc-53876, Santa Cruz Biotechnology;
Immunohistochemistry staining:
CD3, 14-0032-82, Thermo Fisher Scientific,
CD68, ab283654, Abcam,
Ly6G, ab238132, Abcam;
Flow cytometry:
Anti-CD16/CD32 antibody (101301, BioLegend),
Fixable viability dye (eFluor™ 780, 65-0865-14, eBioscience),
CD45 (PE/Cyanine7, 147703, BioLegend),
CD3 (PE, 100205, BioLegend),
F4/80 (FITC, 123107, BioLegend),
CD68 (APC, 137007, Biolegend),
Isotype controls:
PE/Cyanine7 Rat IgG2b, κ, 400617, Biolegend,
PE Rat IgG2b, κ, 400607, Biolegend,
FITC Rat IgG2a, κ, 400505, BioLegend,
APC Rat IgG2a, κ, 400511, Biolegend.

Validation

All antibodies are commercially available. Antibodies employed here in our manuscript were previously reported and routinely used for the application used. All companies used report quality control measures to ensure validity and reproducibility.

Immunofluorescence staining:
cytokeratin 5 antibody (Abcam, ab52635) was validated for IF staining in mice tissue by Abcam https://www.abcam.cn/products/primary-antibodies/cytokeratin-5-antibody-ep1601y-cytoskeleton-marker-ab52635.html,
cytokeratin 10 antibody (Abcam, ab52635) was validated for IF staining in mice tissue by Abcam https://www.abcam.cn/products/primary-antibodies/cytokeratin-10-antibody-ep1607ihcy-cytoskeleton-marker-ab76318.html
cytokeratin 17 antibody (Proteintech, 17516-1-AP) was validated for IF staining by Proteintech https://www.ptgcn.com/products/KRT17-Specific-Antibody-17516-1-AP.htm#product-information
TWIST2 antibody (Proteintech, 66544-1-Ig) was validated for IF staining by Proteintech https://www.ptgcn.com/products/TWIST2-Antibody-66544-1-Ig.htm
Ki67 antibody (Servicebio, GB121141) was validated for IF staining in mice tissue by Servicebio https://www.servicebio.com/goodsdetail?id=46801
SCD1 antibody (Proteintech, 66544-1-Ig) was validated for IF staining by Proteintech https://www.ptgcn.com/products/SCD-Antibody-28678-1-AP.htm
CRABP1 antibody (Cell Signaling, 13163S) was validated for IF staining in mice tissue based on this publication https://www.nature.com/articles/s41467-018-08247-x
MEST antibody (Proteintech, 11118-1-AP) was validated for IF staining in mice tissue based on this publication https://www.nature.com/articles/s41467-018-08247-x
F4/80 antibody (Proteintech, 29414-1-AP) was validated for IF staining in mice tissue by Proteintech https://www.ptgcn.com/products/F4-80-Antibody-29414-1-AP.htm
FOXP3 antibody (Santa Cruz Biotechnology, sc-53876) was validated for IF staining in mice tissue by Santa Cruz Biotechnology https://www.scbt.com/p/foxp3-antibody-2a11g9?requestFrom=search
Immunohistochemistry staining:
CD3 antibody (Thermo Fisher Scientific, 14-0032-82) was validated for IHC staining in mice tissue based on this publication https://www.nature.com/articles/s41467-018-08247-x
CD68 antibody (Abcam, ab283654) was validated for IHC staining in mice tissue by Abcam https://www.abcam.cn/products/primary-antibodies/cd68-antibody-epr23917-164-ab283654.html
Ly6G antibody (Abcam, ab283654) was validated for IHC staining in mice tissue by Abcam https://www.abcam.cn/products/primary-

antibodies/ly6g-antibody-epr22909-135-ab238132.html
Flow cytometry:
Anti-CD16/CD32 antibody (BioLegend, 101301) was validated for Flow Cytometry in mice tissue by BioLegend https://www.biolegend.com/en-us/products/purified-anti-mouse-cd16-32-antibody-190
Fixable viability dye eFluor™ 780 (eBioscience, 65-0865-14) was validated for Flow Cytometry in mice tissue by eBioscience https://www.thermofisher.cn/order/catalog/product/65-0865-14?SID=srch-srp-65-0865-14
PE/Cyanine7 anti-mouse CD45 Antibody (BioLegend, 147703) was validated for Flow Cytometry in mice tissue by BioLegend https://www.biolegend.com/en-us/products/pe-cyanine7-anti-mouse-cd45-antibody-9794
PE anti-mouse CD3 Antibody (BioLegend, 100205) was validated for Flow Cytometry in mice tissue by BioLegend https://www.biolegend.com/en-us/products/pe-anti-mouse-cd3-antibody-47
FITC anti-mouse F4/80 Antibody (BioLegend, 123107) was validated for Flow Cytometry in mice tissue by BioLegend https://www.biolegend.com/en-us/products/fitc-anti-mouse-f4-80-antibody-4067
APC anti-mouse CD68 Antibody (BioLegend, 137007) was validated for Flow Cytometry in mice tissue by BioLegend https://www.biolegend.com/en-us/products/apc-anti-mouse-cd68-antibody-6600
PE/Cyanine7 Rat IgG2b, κ Isotype Ctrl Antibody (BioLegend, 400617) was validated for Flow Cytometry in mice tissue by BioLegend https://www.biolegend.com/en-us/products/pe-cyanine7-rat-igg2b-kappa-isotype-ctrl-1936
PE Rat IgG2b, κ Isotype Ctrl Antibody (BioLegend, 400607) was validated for Flow Cytometry in mice tissue by BioLegend https://www.biolegend.com/en-us/products/pe-rat-igg2b-kappa-isotype-ctrl-1856
FITC Rat IgG2a, κ Isotype Ctrl Antibody (BioLegend, 400505) was validated for Flow Cytometry in mice tissue by BioLegend https://www.biolegend.com/en-us/products/fitc-rat-igg2a-kappa-isotype-ctrl-1841
FITC Rat IgG2a, κ Isotype Ctrl Antibody (BioLegend, 400511) was validated for Flow Cytometry in mice tissue by BioLegend https://www.biolegend.com/en-us/products/apc-rat-igg2a-kappa-isotype-ctrl-1838

Animals and other research organisms

Policy information about studies involving animals; ARRIVE guidelines recommended for reporting animal research, and Sex and Gender in Research

Laboratory animals: Wild type C57BL/6 animals (Dossy Experimental Animals Co., Ltd.) and Rag2-/- animals on a C57BL/6 background (Shanghai Model Organisms Center Inc.) were used at the age of 6-8 weeks. The mice were housed under standard conditions including temperature of 21-27 °C, humidity of 40-70%, and a 12 h light-dark cycle with free access to food.

Wild animals: The study did not involve wild animals.

Reporting on sex: This study only included female mice, and we did not aim to find sex-dependent differences. According to the reference and results of the preliminary experiment, there was no statistical difference in the healing between male and female mice. Given that male mice are more likely to bite the silicone loop when housed together, we used female mice to attenuate this effect.

Field-collected samples: The study did not involve samples collected from the field.

Ethics oversight: All procedures involving animals were approved by the Institution Review Board of West China Hospital of Stomatology (No. WCHSIRB-D-2020-385).

Note that full information on the approval of the study protocol must also be provided in the manuscript.

Flow Cytometry

Plots

Confirm that:

☒ The axis labels state the marker and fluorochrome used (e.g. CD4-FITC).

☒ The axis scales are clearly visible. Include numbers along axes only for bottom left plot of group (a 'group' is an analysis of identical markers).

☒ All plots are contour plots with outliers or pseudocolor plots.

☒ A numerical value for number of cells or percentage (with statistics) is provided.

Methodology

Sample preparation: The wound tissues were firstly digested by the Epidermis Dissociation Kit (Epidermis Dissociation Kit, mouse; Miltenyi Biotec) for enzymatic epidermal-dermal separation. The epidermis part was dissociated by a gentleMACS Dissociator (Miltenyi), then filtered (70-mm cell strainer, Corning, Durham), centrifuged (300g, 10 min, 4°C), and resuspended with phosphate-buffered saline (PBS) containing 0.5% bovine serum albumin (BSA). The dermis part was cut into 1 mm width pieces and mixed with mixed enzyme solution containing type I collagenase (Gibco, Grand Island) and trypsin (Gibco, Canada), then dissociated by gentleMACS Dissociator (Miltenyi), and digested for 2.5 hours in a hybridization oven (Peqlab PerfectBlot). After being dissociated, filtered, centrifuged, and resuspended in red blood cell lysis buffer (Solarbio), the dermis cells were mixed with epidermis part. Then the dead cells and debris were removed by Dead Cell Removal MicroBeads (Miltenyi).
Single cells digested from skin wound were pre-incubated with purified anti-CD16/CD32 antibody (101301, BioLegend) (1.0μg per 106 cells in 100 μl volume) for 5 to 10 min to block Fc receptors. The cell suspensions were then co-incubated with fixable viability dye (eFluor™ 780, 65-0865-14, eBioscience) and antibodies against surface markers CD45 (PE/Cyanine7, 147703, BioLegend), CD3 (PE, 100205, BioLegend), and F4/80 (FITC, 123107, BioLegend) at 1:400 dilution for 30 min at 4°C in

	the dark (100 µl per antibody per sample). After fixation and permeabilization, cells were then incubated with antibody against intracellular marker CD68 (APC, 137007, Biolegend) at 1:400 dilution for 20 min at 4°C in the dark (100 µl per antibody per sample). Isotype controls of CD45 (PE/Cyanine7 Rat IgG2b, κ, 400617, Biolegend), CD3 (PE Rat IgG2b, κ, 400607, Biolegend), F4/80 (FITC Rat IgG2a, κ, 400505, BioLegend) and CD68 (APC Rat IgG2a, κ, 400511, Biolegend) were used in same concentration. Flow cytometry analysis was performed using Attune Nxt flow cytometer (Thermo Fisher Scientific) and FlowJo 10.8.1. The experiments were performed three times independently (n = 3).
Instrument	Attune NxT Flow Cytometer
Software	FlowJo 10.8.1.
Cell population abundance	Data are provided as percent of CD45+CD3+ and CD45+CD68+F4/80+ cells
Gating strategy	The gating strategies are provided in the supplementary profiles.

☒ Tick this box to confirm that a figure exemplifying the gating strategy is provided in the Supplementary Information.
